# Immunological effects of CD19.CAR-T cell therapy in systemic sclerosis: an extended case study

**DOI:** 10.1186/s13075-024-03451-1

**Published:** 2024-12-13

**Authors:** Maren Claus, Merle Freitag, Meike Ewald, Lea Rodon, Franca Deicher, Carsten Watzl, Philipp Kolb, Hanns-Martin Lorenz, Michael Schmitt, Wolfgang Merkt

**Affiliations:** 1 Department of Hematology, Oncology and Rheumatology, Internal Medicine V, University Hospital Heidelberg, Im Neuenheimer Feld 410, 69120 Heidelberg, Germany; 2 Leibniz Research Center for Working Environment and Human Factors at TU Dortmund (IfADo), Ardeystrasse 67, 44139 Dortmund, Germany; 3 Hiller Research Center, University Hospital Düsseldorf, Medical Faculty of Heinrich-Heine University, Moorenstrasse 5, 40225 Düsseldorf, Germany; 4 Institute of Virology, University Medical Center, Hermann-Herder-Str. 11, 79104 Freiburg, Germany; 5 Faculty of Medicine, Albert-Ludwigs-University, Breisacher Strasse 153, 79110 Freiburg, Germany; 6 Department of Rheumatology, University Hospital Düsseldorf, Medical Faculty of Heinrich-Heine University, Düsseldorf, Germany

**Keywords:** Systemic sclerosis, Pulmonary fibrosis, CAR-T cell therapy, NK cells, NKG2A, Fcγ receptor

## Abstract

**Objective:**

The high potential of CD19.CAR-T cells to treat autoimmune diseases such as Systemic Sclerosis (SSc) supposedly relies on the disappearance of autoantibodies. Here we investigated effects of CAR-T cells on the innate immune system which is an important contributor to pathology in SSc.

**Methods:**

Longitudinal analysis of peripheral blood mononuclear cells from an Scl70 + SSc patient treated with CAR-T cells sampled over 18 months by 29-color spectral flow cytometry, in vitro experiments using sera from patient cohorts.

**Results:**

In the patient treated with CAR-T cells, the substantial clinical improvement was paralleled by dynamic changes in innate lymphoid cells, namely Fcγ-receptor IIIA-expressing natural killer (NK) cells. NK cells adopted a more juvenile, less activated, and less differentiated phenotype. In parallel, the potency of serum to form Scl70-containing immune complexes that activate Fcγ-receptor IIIA decreased over time. These observations suggested a mechanistic link between reversal of adaptive autoimmunity and recovering Fcγ-receptor IIIA-expressing innate immune cells after CAR-T cell therapy via regressing immune complex activity. Experiments with sera from the non-CAR-T-treated SSc cohort confirmed that Scl70-containing immune complexes activate Fcγ-receptor IIIA-expressing NK cells in a dose-dependent manner, substantiating the relevance of this link between adaptive and innate immunity in SSc.

**Conclusion:**

This report describes for the first time the phenotypic recovery of innate Fcγ-receptor-expressing cells in an SSc patient treated with CAR-T cells. Decreasing autoantibody levels associated with a reduced ability to form functional immune complexes, the latter appearing to contribute to pathology in SSc via activation of Fcγ receptor IIIA + cells such as NK cells.

**Graphical Abstract:**

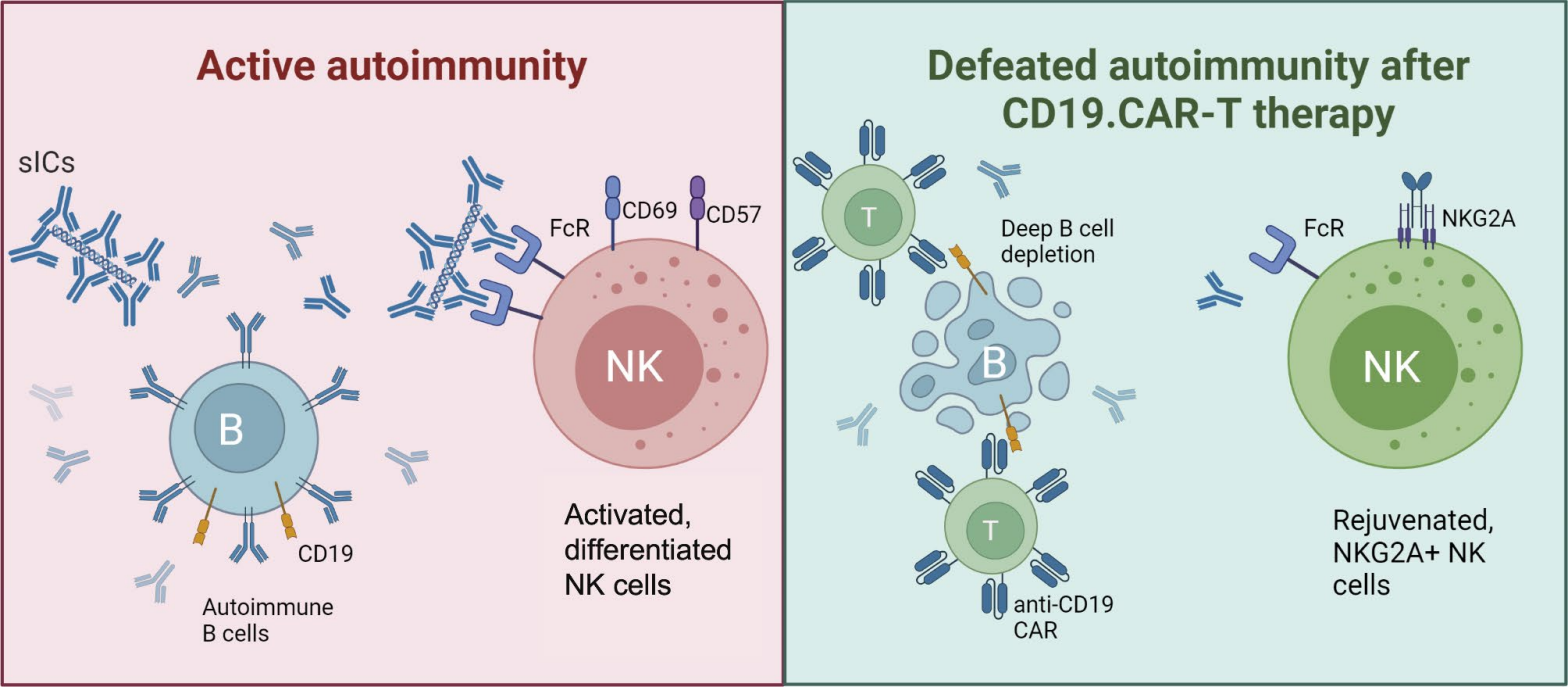

Proposed mechanism of involvement of NK cells and soluble Immune Complexes (sICs) in disease progression during active autoimmunity in SSc (left) and resolution of fibrosis after deep B cell depletion by CD19.CAR-T cells and disappearance of autoantibodies (right).

**Supplementary Information:**

The online version contains supplementary material available at 10.1186/s13075-024-03451-1.

## Background

Standard therapy of Systemic Sclerosis (SSc)-associated interstitial lung disease (ILD), an autoimmune disease with often fatal prognosis, at best decelerates progression. As published earlier, we recently treated an Scl70 + SSc patient with rapidly progressing ILD (NSIP pattern) for the first time with 3^rd^ generation CD19.CAR-T cells [1]. Within 6 months, lung function improved substantially and pulmonary fibrosis regressed. The amelioration of pulmonary fibrosis was defined as improvements of clinical parameters in analogy to the definition of progressive pulmonary fibrosis (PPF), including improved dyspnea, diffusing capacity for carbon monoxide and CT findings [2]. Regressing fibroblast pathology as a key factor in fibrosis was substantiated by slowly regressing signals in *fibroblast activation protein*-inhibitor-based (FAPI)-PET/CT imaging [1]. The substantial clinical improvement lasted until the last follow up 11 months after CAR-T therapy. In addition, the Rodnan skin score as a standard readout of skin fibrosis halfened and markers of chronic autoimmunity including circulating immune complexes that could trigger effects via the Fcγ-receptors CD16 and CD32 normalized. During the observation period, no severe infectious side effects occurred.

This case offered the unique opportunity to investigate the immune landscape in a phase of regressing autoimmune PPF. On the search for a better understanding of autoimmunity and treatment response, we had sampled peripheral blood mononuclear cells (PBMCs) over 18 months, allowing for longitudinal measurements. We hypothesized that the disappearance of pathogenic autoantibodies and antigen-containing immune complexes after CAR-T cell therapy [1, 3] may have secondary effects on Fcγ-receptor bearing immune cells. As Natural Killer (NK) cells are controlled by the Fcγ-receptor CD16 in contrast to B and T cells and as NK cells have recently been associated with pulmonary fibrosis, we were especially interested in these innate lymphoid cells [4, 5].

## Methods

### Patients and cells

The local institutional review boards approved this study (University of Heidelberg, S-272/2021). The CAR-T cell patient, SSc patients, SLE (Systemic Lupus Erythematosus) patients and healthy donors gave their written informed consent to the therapeutic procedures, blood donations and publication. Venous blood was collected in heparinized tubes and peripheral blood mononuclear cells (PBMCs) were isolated at distinct time points. PBMCs were cryopreserved until analysis. The five SSc patients presented in Fig. 3C were all suffering from severe Scl70 + diffuse cutaneous SSc. The mean disease duration was 8.6 years (range 2–22). 4/5 patients had ILD. The mean modified Rodnan Skin Score was 26/51 (range 8–46). Only one of the patients was on rituximab at the time of blood donations, two patients received no immunosuppressive drugs, one received tocilizumab + nintedanib and the last patient was on mycophenolate and 5 mg prednisone.

### Quantification of bioactivity of Scl70 immune complexes in serum of SSc and SLE patients

Recombinant Scl70 recombinant protein was purchased from Sigma. Mouse BW5147 reporter cells expressing the chimeric human Fc receptor CD16 were described earlier [6]. These cells secrete human IL-2 upon stimulation of the respective chimeric Fc receptor. BW5147-CD16 reporter cells were incubated for 16 h with diluted patient serum (1:100 in RPMI / 10% FCS) in the absence or presence of the indicated concentrations of Scl70 antigen. Samples without patient serum / Scl70 or treated with immobilized IVIg (Octagam, Octapharma) served as negative control and positive controls, respectively. Reporter cell activity was measured as production of human IL-2 upon stimulation of CD16 by immune complexes. Concentration of hIL-2 in culture supernatant was quantified by ELISA according to the manufacturer’s instructions.

### Functional analysis of PBMCs and isolated NK cells

Healthy volunteer-derived PBMCs were treated for 16 h with diluted patient sera (1:100 in RPMI + 10% FCS) in the absence or presence of the indicated concentrations of Scl70 antigen. After stimulation, cells were stained for CD3, CD56, CD69, and Annexin V served as dead cell exclusion marker. Cells were analyzed by flow cytometry on a BD LSRII. Healthy NK cells were freshly isolated using a negative isolation strategy based on magnetic beads, as per protocol from the manufacturer (Mojo Sort Human NK cell isolation kit, Biolegend). Degranulation was measured by co-culturing isolated NK cells with the anti-CD107a PeCy5 antibody from BD Biosciences overnight and flow cytometry.

### Spectral flow cytometry

Thawed PBMCs were stained with a dead cell exclusion marker, washed and stained using a cocktail of 28 antibodies for 20 min at 4 °C in the dark. True-Stain Monocyte Blocker™ (Biolegend) was applied. The panel contained an antibody detecting the CD19.CAR (Miltenyi), cell lineage markers (CD3, CD16, CD56, CD4, CD45, CD19, CD8a, CD14), antibodies targeting CD27, CD38, CD161, CD57, CD66b, CD18, CD69, CD16, CD32A, CD32B/C, CD64, as well as KLRG1, HLA-DR, NKp44, TIGIT, NKG2A, NKG2C, TRAIL and PD1 (from BD or Biolegend)(supplementary Table S1). After washing, cells were analyzed immediately on a Cytek^®^ Aurora. Data were analyzed using FlowJo (versions 10.8.2 and 10.9; FlowJo LLC, USA) incl. the plugins DownSample, tSNE (optSNE), Phenograph and Cluster Explorer for high parameter analysis.

To eliminate day-to-day variation and batch effects, we performed stainings of all samples on the same day with the same antibody mastermix. NK cells were pre-gated based on live lymphocytes and their expression of CD56 and lack of CD3. UMAP analysis of PBMC was done with the plugin for FlowJo with default settings on all parameters (supplementary Table S1), UMAP analysis of pre-gated NK cells was performed on the parameters listed below. For tSNE analysis, we used the default settings automatically chosen by the optSNE module of FlowJo 10.9 software. Phenograph analysis was run with version 3.0 plugin for FlowJo with default settings and k = 30. For these analyses, the following parameters were included: CD32B/C; CD8; PD-1; CD18; CD38; NKG2C; CD16; CD161; CD56; CD57; KLRG1; HLA-DR; NKp44; CD69; TIGIT; NKG2A; TRAIL; CTLA-4; CD27; CD32A.

### Statistical analysis

The Friedman test was applied to compare (> 2) paired groups including Dunn’s post test. To compare two groups, Wilcoxon paired one-tailed test was applied. In graphs, * represents *p* < 0.05 and ** represents *p* < 0.01.

## Results

**Fig. 1:**
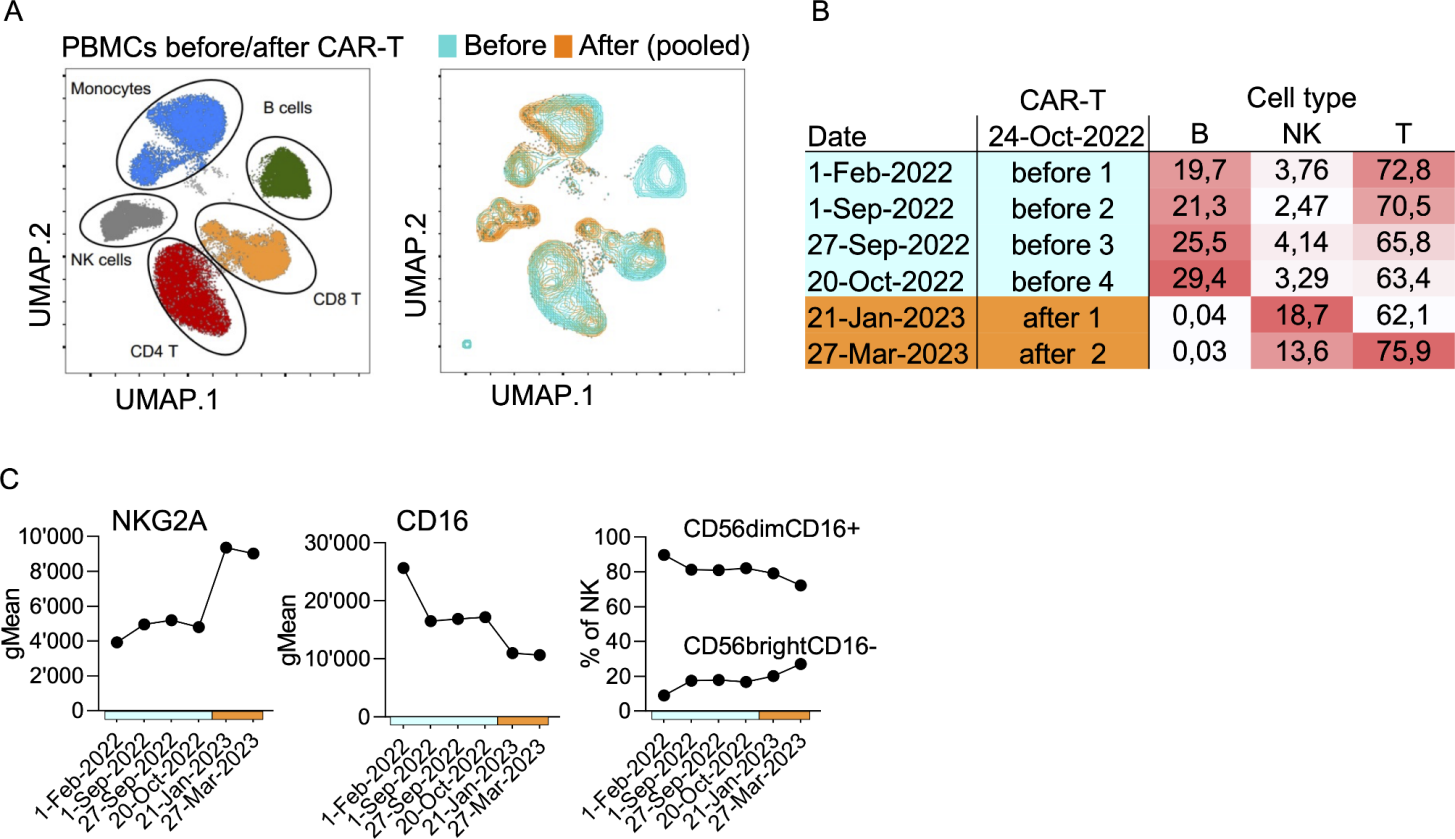
Shifts in Lymphocyte populations during the treatment course in the case study. Thawed PBMCs from the SSc patient treated with CD19.CAR-T cells were analyzed by spectral flow cytometry. **A** UMAPs of cell types within PBMCs based on high-dimensional spectral flow cytometry test results. The right UMAP shows concatenated PBMC samples from timepoints before vs. after CAR-T cell infusion. **B** Lymphocyte subset proportions were determined using manual standard gating strategies. **C** The expression profiles of all surface markers included in the 29-parameter panel were analyzed on total NK cells. Except for CD16 and NKG2A that are shown in the graph, no clear tendencies could be observed during the treatment course. On the right, manually gated major NK subsets (CD56^dim^CD16 + vs. CD56^bright^CD16-) are shown

Except for B cells, spectral flow cytometry analysis revealed the presence of all important PBMC cell types, with CAR-T therapy-associated shifts in each cell type indicating complex cellular changes (Fig. 1a). The most striking difference was a strong relative increase of NK cells after CAR-T cell infusion. While NK cell percentages were at the lower limit of normal before CAR-T therapy, NK cells increased 4–6 fold thereafter, thus filling the therapy-associated lymphopenic gap (Fig. 1b, supplementary Fig. S1). Analyzing protein expression on total NK cells, only few trends were observed (Fig. 1c). The most prominently altered protein was NKG2A. This inhibitory receptor was two-fold increased after CAR-T therapy. According to the literature, NKG2A is expressed on immature CD56^bright^ NK cells and less mature CD56^dim^ NK cells, but has also been discussed as marker for exhausted NK cells [7–9]. In addition, total CD16 gradually decreased during the disease/treatment course (Fig. 1c). Classic NK subsets (CD56^bright^ vs. CD56^dim^) showed a slight increase of CD56^bright^ cells over time, which incompletely explained the changes of NKG2A and CD16.

**Fig. 2:**
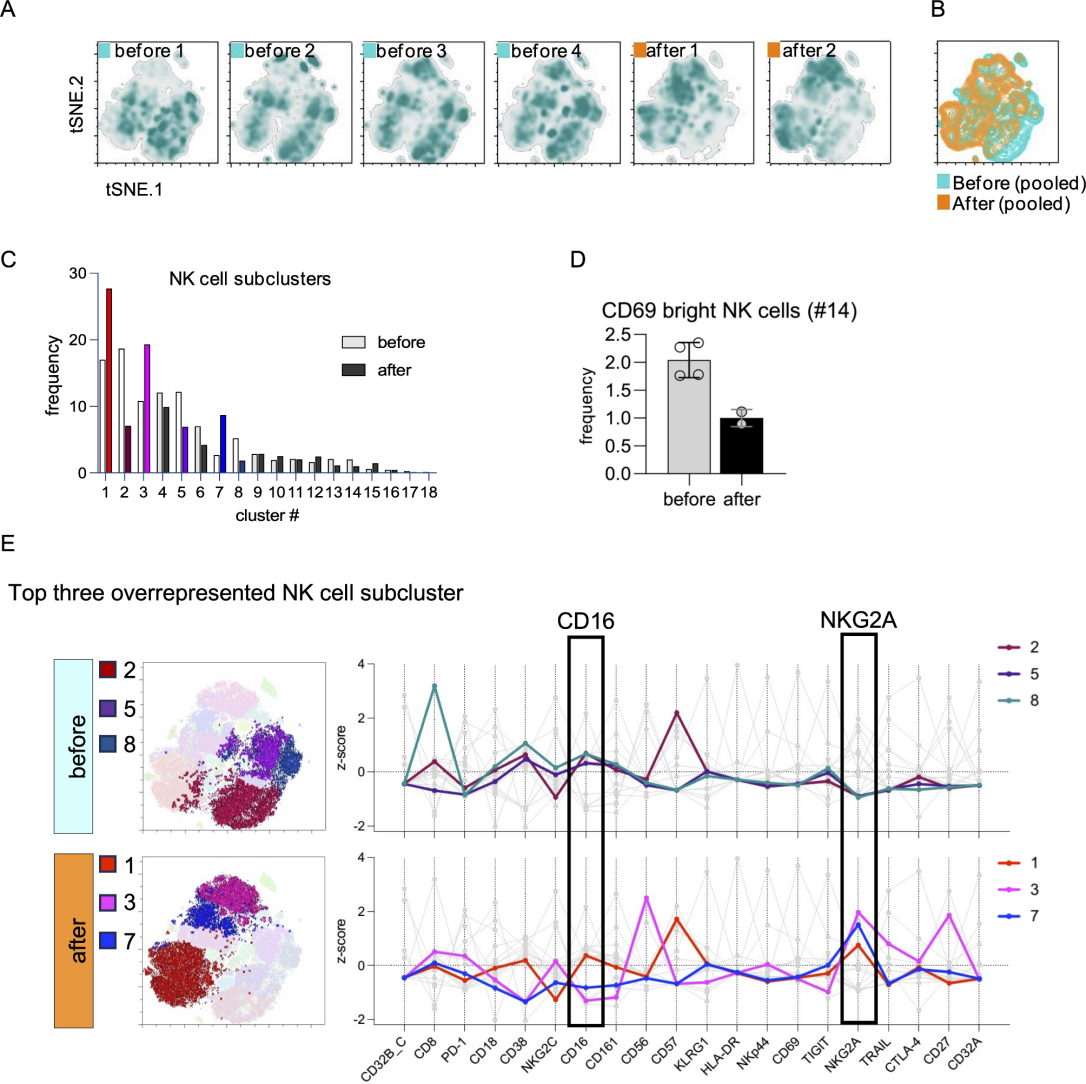
High-dimensional analysis of NK cell dynamics during the treatment course. Thawed PBMCs were analyzed by spectral flow cytometry as shown in Fig. 1. Analyses of pre-gated NK cells are shown. **A** tSNE was used for unbiased dimensionality reduction of NK cells; the graph shows individual time points. **B** tSNE overlay of concatenated samples from before and after CAR-T cell therapy shown in panel A. **C** AI-sustained unbiased Phenograph cluster exploration yielded in 18 NK cell subclusters. The bar diagram compares samples before and after CAR-T therapy within each cluster. Filling of bars denotes after CAR-T therapy and colored bars represent clusters overrepresented before and after CAR-T cell therapy, respectively. The same color-code is used in E. **D** Frequencies of NK cell subcluster #14 characterized by a bright expression of the lymphocyte activation marker CD69. **E** Analysis the top three overrepresented subclusters before (top) and after (bottom) CAR-T cell therapy. Top three subclusters were visually identified within the ten most frequent NK cell subclusters. All top three overrepresented NK cell subclusters after CAR-T therapy were characterized by strong expression of NKG2A, an inhibitory receptor and marker of early developmental stages in NK cells. Subcluster #3 corresponds to CD56^bright^CD16- NK cells, which are believed to be precursors of more mature CD56^dim^CD16+ NK cells. Subcluster #7 is an intermediate stage being NKG2A^bright^ and CD16 positive, but not CD16^bright^. Given the overall expression profile of this subset, these “CD56^dim^CD16^dim^NKG2A^bright^CD57^neg^” NK cells most likely represent less mature, juvenile NK cells. CD16+ CD57^bright^ NK cell subcluster #2 represents terminally differentiated NK cells

Unbiased analysis using tSNE revealed clear shifts in the NK cell compartment (Fig. 2a, b) and Phenograph clustering resulted in 18 NK subclusters (Fig. 2c). A longitudinal analysis of these subclusters showed shifts over time (Fig. 2c). We found that subcluster #14, characterized by a high expression of the activation marker CD69, was about halved in numbers (Fig. 2d), indicating less NK cell activation. An analysis of the top three overrepresented subclusters before vs. after CAR-T therapy revealed that NKG2A was generally increased after CAR-T therapy (Fig. 2e, supplementary Fig. S2). CAR-T therapy was associated with an increase of subcluster #3 representing developmentally early CD56^bright^CD16^low^ (CD57^low^NKG2A^bright^) cells and being one of the most prevalent subsets post treatment. In addition, CAR-T therapy was associated with an increase of subcluster #7 characterized by an intermediate expression of CD16. Given the overall expression profile of this subcluster, these “CD56^dim^CD16^intermediate^NKG2A^bright^CD57^neg^” NK cells most likely represented less mature, juvenile NK cells (Fig. 2e). We confirmed the presence of CD16^intermediate^ juvenile NK cells using UMAP as an alternative strategy for dimensionality reduction and observed that juvenile NK cells made up about 14% of total NK cells after, in contrast to about 7% before CAR-T therapy (not shown). In line, terminally differentiated CD57^bright^ NK cells (#2) declined after CAR-T therapy.

Thus, CAR-T therapy was associated with a profound relative increase of NK cells. Beyond this, altered NK phenotypes indicated an active process beyond purely relative changes secondary to the directly therapy-related B cell count decrease. The NK cell pool showed phenotypic signs of rejuvenation as well as lower degrees of activation and terminal differentiation. An over-representation of NKG2A-expressing NK cells characterized the phase of lung fibrosis resolution.

**Fig. 3:**
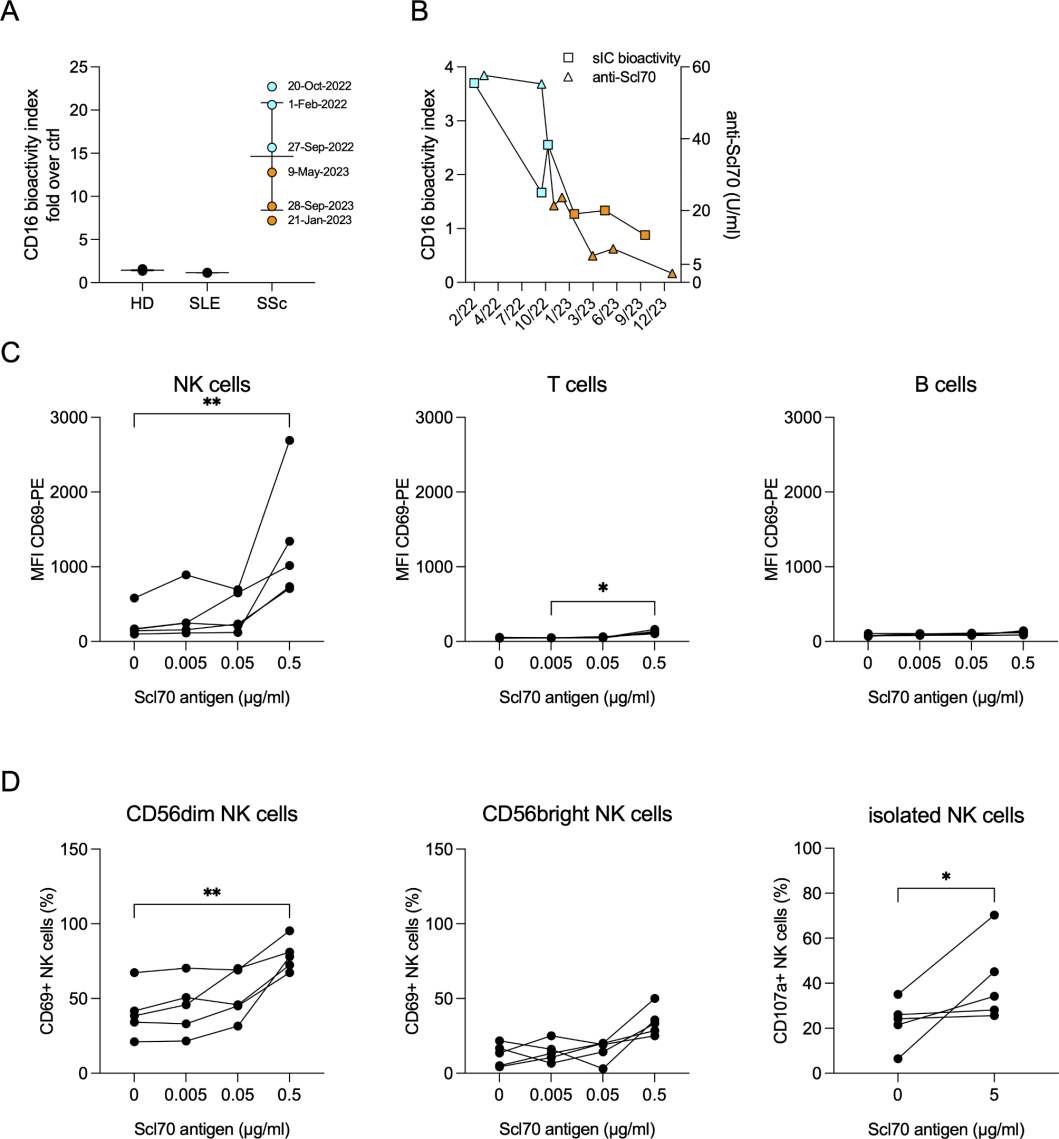
Scl70 antigen and anti-Scl70 autoantibodies form soluble immune complexes (sICs) that bind and activate CD16 on NK cells. **A** Bioactivity of sICs in serum from the described CAR-T cell patient, SLE patients (*n* = 3) and healthy controls (*n* = 4) in the presence of 0.05 µg/ml Scl70 antigen. Data are shown as mean ± SEM and are presented as fold over CD16 bioactivity in the absence of Scl70 antigen. For the CAR-T cell patient, serum samples before (blue) and after (orange) CAR-T cell treatment are shown. **B** Longitudinal measurement of CD16 activation by sICs (squares) in the presence of 0.05 µg/ml Scl70 antigen before (blue) and after (orange) CAR-T cell treatment and serum anti-Scl70 autoantibody concentration (triangles). Anti-Scl70 values below 5 U/ml are considered negative. **C** PBMC were stimulated with serum from 5 different Scl70 + SSc patients in the presence of the indicated concentrations of Scl70 antigen. Upregulation of the activation marker CD69 of NK cells, B cells and T cells was determined by flow cytometry. **D** left and middle: In the same experiment shown in (C), CD56^dim^ and CD56^bright^ NK cells were gated separately. Shown is the frequency of CD69-positive cells as a technical confirmation of activation of Fc-receptor-bearing CD56^dim^ NK cells by Scl70-containing immune complexes. Right; from the same PBMCs, NK cells were in parallel isolated and their degranulation without (0) and with (5 µg/ml) Scl70 antigen in the same five sera from Scl70 + SSc patients as shown in **C** and **D** left/middle was determined. CD107a + NK cells represent degranulated NK cells, expressed as percentage of all NK cells. These data confirm functionally relevant activation of NK cells by Scl70-containing immune complexes

These phenotypic differences in progressive vs. resolving autoimmunity - i.e. before vs. after CD19.CAR-T cell therapy - in our patient imply mechanisms that activate NK cells in active autoimmunity. These mechanisms likely involve B cells and/or their pathogenic products, namely autoantibodies. In order to mimic local inflammatory processes, we added Scl70 antigen to the patient’s sera and detected formation of immune complexes that activate CD16 using a novel Fc receptor reporter cell assay [6] (Fig. 3a). Scl70 antigen did not induce immune complexes in healthy and SLE control sera. The analysis of our CAR-T-treated SSc patient’s sera from different timepoints indicated that earlier timepoints tended to form soluble immune complexes with a higher potency to activate CD16 than later timepoints (Fig. 3a, b). Of note, in other SSc cases treated with a short-lived 2^nd^ generation CAR-T cells, Scl70 autoantibodies did not disappear after treatment [10]. In contrast, in our patient having received a different treatment regimen with 3^rd^ generation CAR-T cells that persisted during the follow up period, treatment was associated with a slowly degrading and finally vanishing Scl70 autoantibody only after a long period of 15 months of persisting CAR-T cells (Fig. 3b) and Merkt et al. [11]. In the reporter assay, the course of CD16 bioactivity was paralleled by decreasing levels of Scl70-autoantibody over time, suggesting dose-dependent effects (Fig. 3b). Finally, Scl70 antigen added to sera from five different Scl70 + SSc patients led to a dose-dependent activation of NK cells as measured by increased CD69 expression levels, but not B cells that lack activating Fc receptors such as CD16 (Fig. 3c). These data were confirmed by showing that Scl70-containing immune complexes significantly enhanced the frequency of CD69-positive cells on the Fcγ-receptor-bearing CD56^dim^ NK cells but not on Fcγ-receptor-negative CD56^bright^ NK cells (Fig. 3d). In addition, degranulation of freshly isolated NK cells was significantly higher in CD107a assays in presence of Scl70-containing immune complexes (Fig. 3d, right graph). T cells showed a minor yet significant increase in CD69 at high concentrations of Scl70 (Fig. 3c), which may reflect a small T cell subset expressing CD16 [12] or indirect effects, e.g. via cytokines secreted from Fcγ-receptor bearing cells.

Together, these data provide initial evidence that Scl70 autoantibodies in conjunction with their autoantigen mediate NK cell activation in active SSc and that the therapeutic removal of autoantibodies mediates NK cell recovery.

## Discussion

Even though being a single case study, the finding that CAR-T therapy associated with a rejuvenation of CD16^dim^ NK cells and, at the same time, associated with the disappearance of CD16-activating circulating immune complexes [1] may be of great interest, given the emerging role of CD16-activating circulating immune complexes and CD16 + NK cells in SSc and ILD [4, 5, 13, 14]. Antibody-dependent cellular cytotoxicity by CD16 + NK cells has been proposed as a mechanism how autoantibodies and circulating immune complexes may damage endothelial cells in SSc [15]. Interestingly, CD16 variants were associated with the emergence of Scl70 autoantibodies and ILD in SSc [16], and the downstream signaling chain of CD16, CD247, is a risk locus for SSc [17]. These correlative data together provide strong evidence for a role of CD16-binding immune complexes and CD16 signaling in the pathophysiology of Scl70 + SSc and autoimmune pulmonary fibrosis. Our finding that the Scl70 antigen leads to CD16-activating immune complexes in sera from our SSc cohort confirms a mechanistic link of Scl70-autoantibodies, CD16 and NK cells. In line, several recent transcriptome studies implicated CD16 + NK cells in the pathogenesis of SSc [4, 5, 14].

The association of clinical response to therapy with increased NK cells as well as their phenotypic changes suggests a role of NK cells in this condition and/or in its treatment. A protective role of NK cells in fibrotic diseases has been suggested given their physiological role in controlling myofibroblasts at the end of wound healing [18]. We suggest that NK cells in combination with immune complexes may be rather pathogenic via CD16-induced tissue injury, while in a state of cleared immune complexes, other NK cell functions may dominate, perhaps natural cytotoxicity towards activated myofibroblasts limiting or even resolving fibrosis [18].

## Conclusion

In summary, our preliminary finding that NK cells gained a more juvenile, less activated, and less differentiated phenotype during the resolution of fibrosis suggests that CD19.CAR-T cells possessed positive effects on this part of pathophysiology. While it warrants further study for confirmation, this report suggests pathogenicity of Scl70 autoantibodies in SSc and is the first to describe the recovery of FcγR-expressing NK cells by CD19.CAR-T cell-containing immunosuppressive therapy in autoimmune PPF.

## Electronic supplementary material

Below is the link to the electronic supplementary material.

Supplementary Material 1

Supplementary Material 2

## Data Availability

The raw data supporting the conclusions of this article will be made available from the corresponding author on reasonable request.

## Abbreviations

CAR: Chimeric antigen receptor
SSc: Systemic sclerosis
ILD: Interstitial lung disease
PPF: Progressive pulmonary fibrosis
FAPI: Fibroblast activation protein-inhibitor-based
PBMC: Peripheral blood mononuclear cells
NK cells: Natural Killer cells
SLE: Systemic Lupus Erythematosus
tSNE: t-Distributed Stochastic Neighbor Embedding
UMAP: Uniform Manifold Approximation and Projection
sICs: soluble Immune Complexes
