# Supplementary material for: Immunological effects of CD19.CAR-T cell therapy in systemic sclerosis: an extended case study
Source: Arthritis Res Ther. 2024 Dec 13;26:211. doi: 10.1186/s13075-024-03451-1 (PMC11639114; doi:10.1186/s13075-024-03451-1)
Supplement: Supplementary file 1 — Supplementary Material 1 [file 13075_2024_3451_MOESM1_ESM.pdf]

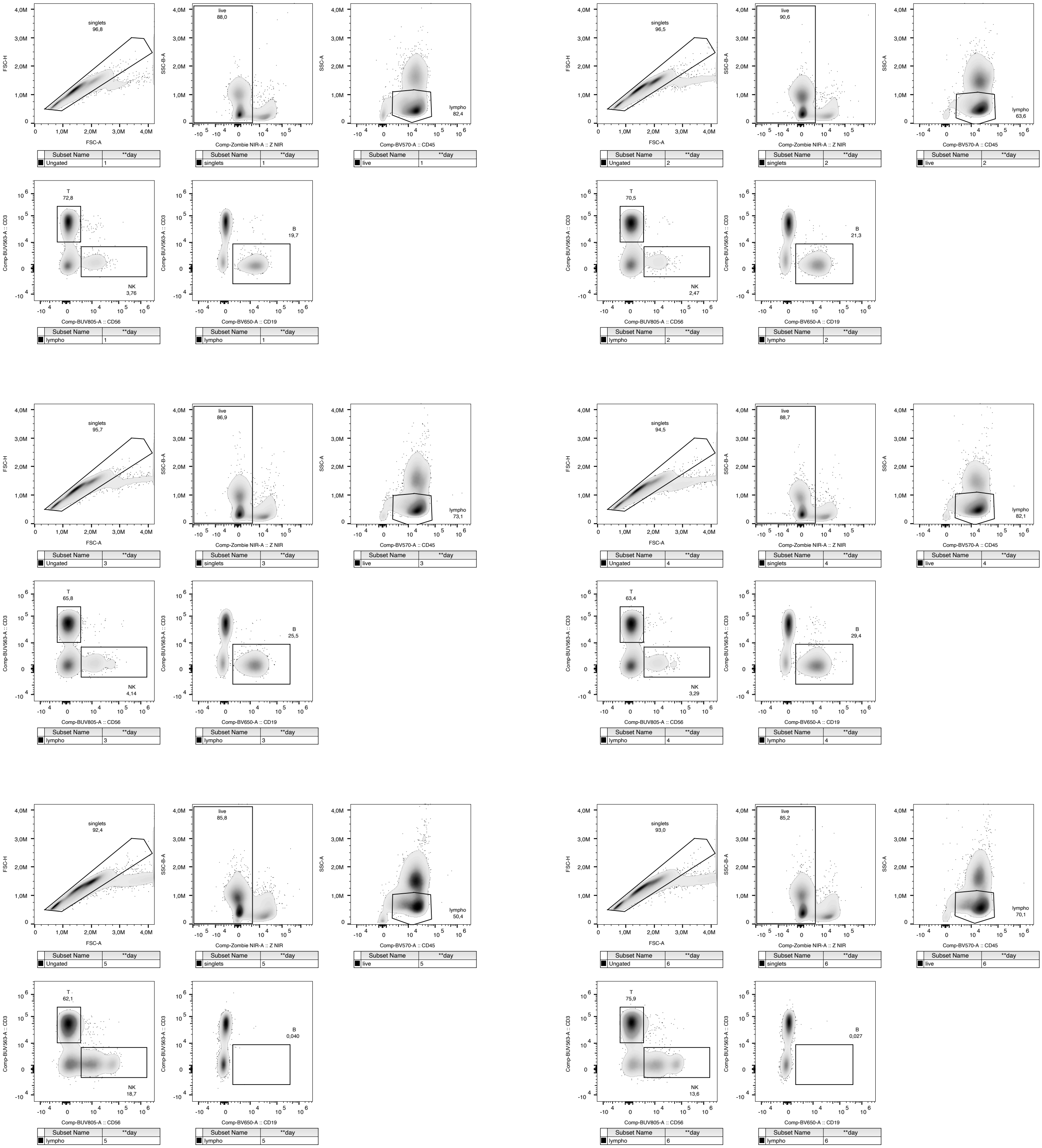

Supplementary Figure 1
Gating strategy for quantification of lymphocyte subsets shown in Fig. 1B

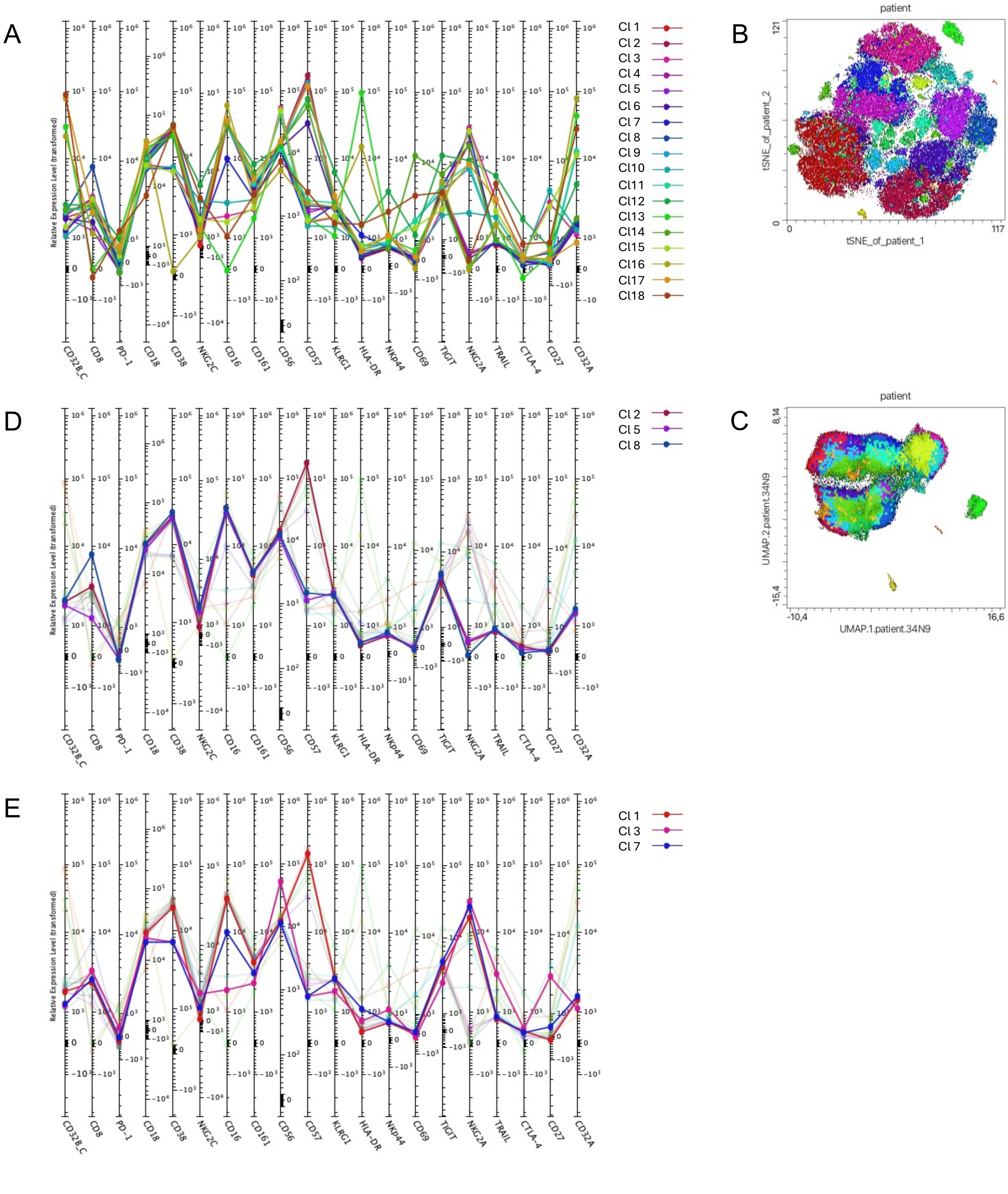

Supplementary Figure 2
(A) Patient NK cells were analyzed using the Phenograph plugin for FlowJo on the depicted parameters. Resulting clusters are shown as overlays on a (B) tSNE (optSNE) and (C) UMAP projection, respectively. Features of (D) clusters 2, 5 and 8 with higher abundance before treatment, and (E) clusters 1, 3 and 7 with higher abundance after treatment are shown.

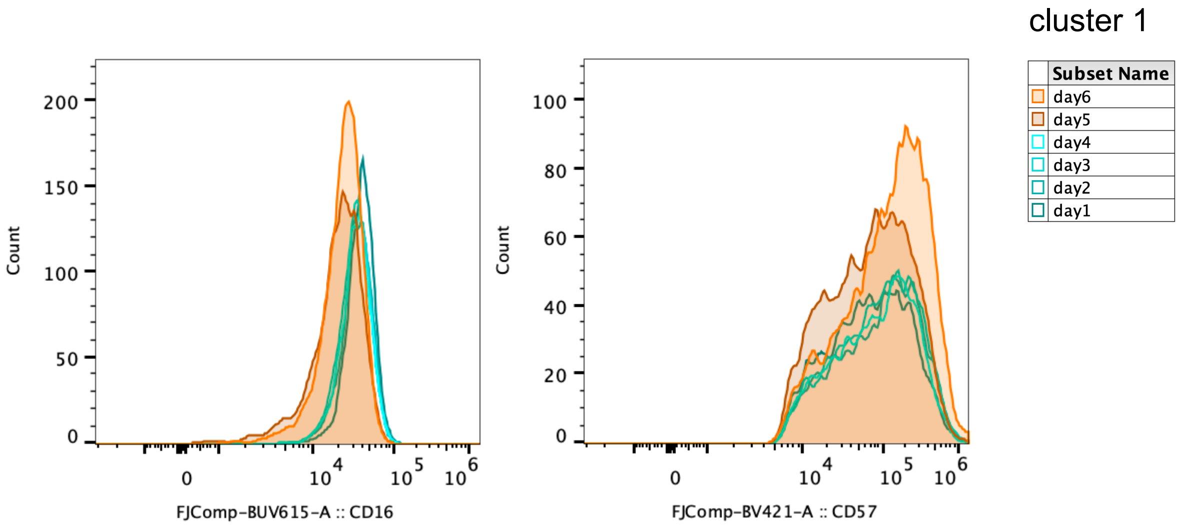

Supplementary Figure 3
Histograms showing kinetics of CD16 and CD57 within cluster 1
