# Supplementary material for: Immunological effects of CD19.CAR-T cell therapy in systemic sclerosis: an extended case study
Source: Arthritis Res Ther. 2024 Dec 13;26:211. doi: 10.1186/s13075-024-03451-1 (PMC11639114; doi:10.1186/s13075-024-03451-1)
Supplement: Supplementary file 2 — Supplementary Material 2 [file 13075_2024_3451_MOESM2_ESM.docx]

**Suppl. Table 1: Antigens, antibody clones and coupled fluorochromes, reagents, distributors and Ab dilution are listed**

| Target | clone | Fluorochrome | Peak Emission Channel | distributor | dilution 1/x |
| --- | --- | --- | --- | --- | --- |
|  |  |  |  |  |  |
| CD38 | HB7 | BUV395 | UV2 | BD Biosciences | 100 |
| CD159c (NKG2C) | 134591 | BUV496 | UV7 | BD Biosciences | 50 |
| CD3 | UCHT1 | BUV563 | UV9 | BD Biosciences | 200 |
| CD16 | 3G8 | BUV615 | UV10 | BD Biosciences | 400 |
| CD161 | HP-3G10 | BUV661 | UV11 | BD Biosciences | 100 |
| CD56 | B159 | BUV805 | UV16 | BD Biosciences | 500 |
| CD57 | HNK-1 | BV421 | V1 | Biolegend | 400 |
| CD4 | RPA-T4 | V450 | V3 | BD Biosciences | 200 |
| CD64 | 10.1 | BV480 | V5 | BD Biosciences | 200 |
| KLRG1 | 2F1 | BV510 | V7 | Biolegend | 50 |
| CD45 | HI30 | BV570 | V8 | Biolegend | 400 |
| HLA-DR | L243 | BV605 | V10 | Biolegend | 50 |
| CD19 | HIB19 | BV650 | V11 | Biolegend | 200 |
| NKp44 | p44-8 | BV711 | V13 | BD Biosciences | 200 |
| CD69 | FN50 | BV750 | V14 | Biolegend | 400 |
| TIGIT | 741182 | BV786 | V15 | BD Biosciences | 100 |
| CD19.CAR-T | REA1297 | Vio® Bright B515 | B1 | Miltenyi Biotec | 25 |
| CD8a | RPA-T8 | AF532 | B3 | ThermoFisher Scientific | 200 |
| CD14 | MφP9 | BB700 | B9 | BD Biosciences | 1000 |
| CD27 | M-T271 | PerCP-Cy5.5 | B9 | BD Biosciences | 50 |
| CD32A | IV.3 | RB780 | B13 | BD Biosciences | 1000 |
| CD159a (NKG2A) | Z199 | PE | YG1 | Beckman Coulter | 100 |
| CD152 (CTLA-4) | BNI3 | PE-Dazzle | YG3 | Biolegend | 100 |
| CD66b | 6/40c | PE-Fire 640 | YG4 | Biolegend | 400 |
| CD253 (TRAIL) | N2B2 | PE-Cy7 | YG9 | Biolegend | 50 |
| CD279 (PD-1) | EH12.1 | AF647 | R2 | BD Biosciences | 100 |
| CD18 | TS1/18 | AF700 | R4 | Biolegend | 800 |
| live / dead | n/a | Zombie NIR | R6 | Biolegend | 700 |
| CD32B/C | S18005H | APC-Cy7 | R7 | Biolegend | 400 |
|  |  |  |  |  |  |
| True-Stain Monocyte Blocker™ |  |  |  | Biolegend |  |
| Brilliant Stain Buffer |  |  |  | BD Biosciences |  |
| Human serum |  |  |  | Pan-Biotech |  |
